# Cognitive impairment experienced by Chinese breast cancer survivors

**DOI:** 10.1038/s41598-023-49524-0

**Published:** 2023-12-14

**Authors:** Dan Chen, Lynette Mackenzie, Syeda Zakia Hossain, Jing-Xin Wang, Ping-Lan Jiang, Yuanxiao Wang, Lanhui Qin, Jun Zhen, Jie Jia

**Affiliations:** 1 Department of Rehabilitation Medicine, Jing’an District Central Hospital of Shanghai, Shanghai, China; 2 Department of Rehabilitation Medicine, Huashan Hospital Fudan University, Shanghai, China; 3 Discipline of Occupational Therapy, School of Health Sciences, Faculty of Medicine and Health, University of Sydney, Sydney, NSW 2006 Australia; 4 Discipline of Behavioural Sciences, School of Health Sciences, Faculty of Medicine and Health, University of Sydney, Sydney, NSW 2006 Australia; 5 Department of Rehabilitation Medicine, Zhengzhou Central Hospital of Zhengzhou University, Zhengzhou, Henan China; 6 Department of Breast Surgery, Xiangya Hospital of Central South University, Changsha, China; 7 Department of Breast Surgery, Yunnan Cancer Hospital, Yunnan, China; 8 Department of Rehabilitation, the People’s Hospital of Guangxi Zhuang Autonomous Region, Nanning, China; 9 Department of Rehabilitation, The Fifth Affiliated Hospital of Sun Yat-Sen University, Zhuhai, Guangdong China

**Keywords:** Cancer, Oncology

## Abstract

To identify cognitive function in Chinese breast cancer survivors. Research questions were: is cognitive function was associated with breast cancer and/or chemotherapy treatment and/or psychological functioning:? and did women with breast cancer experience more cognitive and psychological issues than age-matched women without cancer? Breast cancer survivors with chemotherapy (n = 106, mean age = 50.2 ± 9.5), breast cancer survivors without chemotherapy (n = 100, mean age = 50.5 ± 10.0) and matched healthy controls (n = 96, mean age = 47.9 ± 9.1) completed a battery of cognitive and psychosocial functioning. Demographic characteristics were also collected. The Perceived Cognitive Impairment score for cancer groups was significantly higher than for the healthy group (*p* = 0.04), but not between the cancer groups. Processing speed was significantly slower in the cancer groups than in the healthy group (both *p* < 0.001), but not between the cancer groups. Age, living status and education were significantly associated with the FACT-Cog (all *p* < 0.05). The correlations between the FACT-Cog score and BSI score were strong (r = 0.60 *p* < 0.01), and between the HADS anxiety and depression scales were strong (r = 0.53 and 0.50, *p* < 0.01) but correlations were weaker between performance based cognitive tests and measures of psychological functioning. Breast cancer groups indicated more cognitive impairment and reduced psychological functioning compared to the healthy group. However, there was no differences between the breast cancer groups. Chinese breast cancer survivors experienced excess cognitive impairment not associated with usual ageing. Assessment and intervention to address cognitive impairment should be made available to breast cancer survivors.

## Introduction

Breast cancer has been reported as the most common global cancer accounting for 11.7% of new cases^1^. Breast cancer survival is relatively high although treatments such as surgery, chemotherapy (CT), radiation therapy, endocrine therapy, and specific biological therapies can have side effects that impact on quality of life for breast cancer survivors^2,3^. One of the side effects of breast cancer treatment is cognitive changes^4^. This was first reported towards the end of the 1990s^5^, where cancer patients complained of impaired cognitive function after treatment. Cogntive functions affected include impaired memory, attention, speed of processing, and word finding, prompting researchers to assess cognitive function particularly in breast cancer survivors receiving chemotherapy. Chemotherapy was believed to be the main cause of cognitive impairment in cancer patients. A prospective, longitudinal, national multi-center study conducted by Janelsins et al.^6^ completed FACT-COG evaluations of breast cancer survivors and age-matched non-cancer controls before and after chemotherapy, and at 6-month follow-up, and found that patients with breast cancer who were treated in the community reported more cognitive difficulties up to 6 months after treatment with chemotherapy compared to age-matched noncancer controls. Cognitive difficulties can persist and in a study by Koppelmans et al.^7^, the cognitive performance of breast cancer survivors who had received chemotherapy more than 20 years earlier was still significantly worse than that of healthy controls.

Imaging studies have identified structural brain differences in breast cancer survivors who have had chemotherapy and those who have not, and between breast cancer survivors and healthy controls. Cerebral white matter seemed to be particularly vulnerable to the effects of chemotherapy. For example, abnormal microstructural changes in white-matter tracts concerned with cognition were discovered 10 years after completion of chemotherapy in breast cancer survivors compared to those who did not have chemotherapy^8^.

Cognitive impairment presents as what is often referred to as "chemo brain". However, some prospective studies have found that cognitive impairment may predate chemotherapy. For example, Ahles et al.^9^ showed that 22% and Wefel et al.^10^ found 21% of breast cancer patients had cognitive impairment before chemotherapy, suggesting that the cancer itself, surgery or menopause may be implicated. In another prospective study^11^ the correlation between chemotherapy and cognitive impairment was less clear with different subgroups emerging over the follow-up period of chemotherapy treatment being completed—some with a decline in cognitive function that was persistent or improved, and some with no effects. Authors also found that cognitive deficits were unrelated to anxiety or depression, and that chemotherapy cognitive outcomes were not related to menopausal status. Similarly, McDonald et al.^12^ found that cancer groups had decreased frontal hyperactivation compared with controls at 1 month after completion of chemotherapy. Ahles et al.^13^ analyzed the results of multiple longitudinal studies and proposed two hypotheses. The first is that the biology of cancer may contribute to lower cognitive performance; and the second that common risk factors for breast cancer and cognitive changes may exist^13^. In subsequent studies, cognitive manifestations are often referred to as cancer-related cognitive impairment (CRCI)^14^.

However, most of this evidence has come from developed countries. Breast cancer was the most common cancer and the fifth contributor to cancer mortality among women in China in 2015^15^. Urban areas of China recorded the highest incidence and mortality rates for breast cancer, and the most common type of breast cancer diagnosis was invasive ductal carcinoma. The incidence of breast cancer is expected to continue to rise in China^15^. Only two studies were located that have investigated cognition in Chinese populations of women with breast cancer: Zheng et al.^16^ conducted a prospective study of cognitive functioning in newly diagnosed primary breast cancer survivors taking measurements at 18 months and at 3 years post diagnosis, using a battery of assessments including the Logical Memory Subtest from the Chinese Version of the Wechsler Memory Scale (verbal episodic memory), the Chinese Version of the Category Fluency Test (language/executive function), and the Chinese Version of the Stroop Test (attention/executive function). Results indicated a significant improvement in attention and executive function, immediate memory and delayed memory significantly from 18 to 36 months after breast cancer diagnosis. This study did not measure cognitive functioning prior to 18 months post breast cancer diagnosis, did not include a control group, did not compare participants who received chemotherapy and those who did not, and only included survivors in one geographical area of China. Li^17^ conducted a cross sectional study with breast cancer survivors from one medical centre immediately post surgery using self-reported measurs of cognition and psychological status, and also did not include a control group or comparison group of participants with and without chemotherapy. Neither study used the tools recommended by the International Cognition and Cancer Task Force^18^ which were the Hopkins Verbal Learning Test-Revised (HVLT-R), the Trail Making Test (TMT), and the Controlled Oral Word Association (COWA) test.

Therefore, this study addressed some of the limitations of existing studies by collecting data across China, using a control group, comparing chemotherapy and non-chemotherapy groups and using both self-reported and performance based measures. The aims of this study were (1) to explore the cognitive functioning of Chinese breast cancer survivors within 6 months of diagnosis, (2) to compare cognitive functioning of breast cancer survivors who had chemotherapy, with those who did not have chemotherapy and with aged matched healthy Chinese women, (3) to explore the relationship between cognitive functioning and psychological functioning, and (4) to compare cognitive performance scores from tools using self-report, and tools using performance of cognitive tasks.

## Method

### Study design

**Figure 1:**
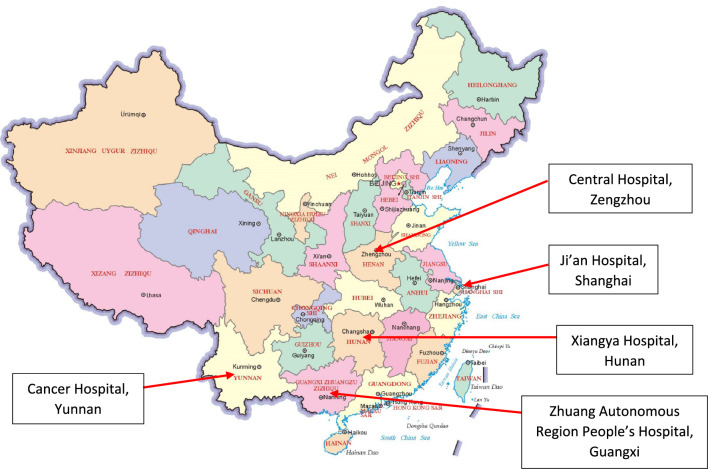
Distribution and location of recruitment centres.

A multicentre, cross-sectional study was conducted that explored the associations between breast cancer treatment with chemotherapy or breast cancer treatment without chemotherapy and CRCI in female breast cancer survivors who had treatment at one of five centres in China. These results were compared with age matched health controls at each centre. The five Chinese centres were, Jing 'an central hospital in Shanghai, Cancer hospital in Yunnan, Central hospital in Zhengzhou, Xiangya hospital in Hunan and Zhuangzu autonomous region people's hospital in Guangxi (Fig. 1).

The participants were recruited from the centres, into three groups. Group A was defined as the breast cancer with chemotherapy group, Group B was defined as the breast cancer with no chemotherapy group, and Group C was defined as the age-matched healthy group. Controls were obtained from the same source population in each centre, and they could be family members or friends of the participants, as well as unrelated participants such as nursing assistants and healthy women recruited from the community. All participants completed the same assessments that included demographic information, cognitive function, and psychological states.

This study was a co-operative study between Huashan hospital affiliated to Fudan university and the University of Sydney. Institutional review boards at the Jing 'an District Central Hospital of Huashan hospital affiliated to Fudan university and the University of Sydney (ID: 2019/722) approved the study before participants enrolled. All research was performed in accordance with relevant guidelinesand regulations, written informed consent was obtained from all participants. Chinese researchers from five centres were trained by two researchers from the University of Sydney (LM and SZH) about how to complete the outcome measures. The training was focused on the assessment of cognitive and psychological functioning.

Women who were due for breast surgery at their respective hospitals were recruited by researchers. To ensure privacy, interested participants were presented with the entire study protocol and the informed consent procedure in a separate meeting room. Recruited participants were asked to sign the informed consent which was reviewed by the investigator. Participants were free to withdraw from the study at any time as highlighted in the informed consent information. Participants were assessed by the same researcher in a separate room throughout the process.

### Study participants

The inclusion criteria for Group A were: (1) Female breast cancer survivors undergoing their first treatment with systemic chemotherapy or had completed it. (2) Between 25 and 75 years of age at time of diagnosis; (3) Fluent in Chinese and able to read and write; (4) Able to understand the content of the study and agrees to participate in this study. The exclusion criteria for Group A were: (1) Diagnosis of a neurodegenerative disease; (2) Use of psychotropic medication; (3) Previous history of cancer; (4) Neurobehavioral risk factors including history of neurological disorder (e.g., Parkinson’s disease, seizure disorder, and dementia); (5) Moderate to severe head trauma.

The inclusion criteria for Group B were: (1) female breast cancer survivors with a first diagnosis before chemotherapy or had not received it; (2) between 25 and 75 years of age at time of diagnosis; (3) fluent in Chinese and able to read and write; (4) Able to understand the content of the study and agrees to participate in the study. These patients were screened with the same exclusion criteria as Group A.

Healthy controls were recruited age matched to the ages of the breast cancer participants. The exclusion criteria for the Group A were adapted to them. All study participants provided written informed consent to participate. The sample size for each group was calculated using the ABS sample calculator. Using the estimation of the total population of breast cancer, an alpha of 0.05% and a confidence level of 95% and *p* =  < 0.001, the sample size should be 75 for each group.

### Demographic and medical information

An investigator-developed questionnaire was used to collect demographic information including age, Body Mass Index (BMI), living status, education, job, and medical information including type of cancer, stage of cancer, radiation therapy status, hormone therapy status, targeted therapy status and any other complications. Medical information was validated by a medical record review by researchers.

### Measures

Eligible participants consented and completed a survey/questionnaire that assessed demographic and medical information, cognitive functioning and psychological status (see supplementary file 1). The instruments used were available and validated for use in both English and Chinese. These were the Functional Assessment of Cancer Therapy-Cognitive Function, FACT-Cog (Version 3)^19^, The Hopkins Verbal Learning Test (HVLT)^20^, The Trail Making Test (TMT)^21^, the Hospital Anxiety and Depression Scale (HADS)^22^ and the Brief Symptom Inventory^23^. Two of the assessments were in-person neuropsychological tests, The Hopkins Verbal Learning Test (HVLT) and the Trail Making Test (TMT), which were administered by trained researchers. The assessments took approximately 40 min to complete.

### Self-reported cognitive measures

The Functional Assessment of Cancer Therapy-Cognitive Function, FACT-Cog (Version 3) was used to measure self-reported cognitive functioning and consists of 37 items designed to assess cognitive complaints. The application of the Chinese version of FACT-Cog has been tested with good reliability and validity^24^. The FACT-Cog measures both cognitive concerns and cognitive abilities, that are considered to be independent of one another. The FACT-Cog Version 3 includes positively expressed sentences (e.g., My memory is as good as it has always been) and negatively expressed sentences (e.g., I have had trouble forming thoughts). The FACT-Cog produces a total score and four subscale scores including:(1) Perceived Cognitive Impairments (CogPCI), (2) Impact of Perceived Cognitive Impairments on Quality of Life (CogQOL), (3) Comments from Others (CogOth), and (4) Perceived Cognitive Abilities (CogPCA). Participants rate items on the CogPCI and CogOth scales on a five-point Likert scale (0 = never to 4 = several times a day) to show the frequency of each event had occurred in the past 7 days. The CogQoL and CogPCA scales are rated on five-point scales (from 0 = not at all to 4 = very much) to show the extent to which each event had occurred in the past 7 days. Positively expressed sentences items (for PCA) are reversed to be calculated, so that lower scores reflect fewer cognitive impairments and better QoL.

### Performance-based tests

Immediate and delayed verbal memory, speed of processing, and executive function were measured using the Hopkins Verbal Learning Test (HVLT) and the Trail Making Test (TMT).

The Hopkins Verbal Learning Test (HVLT) has six different forms that each consist of a 12-item word list, composed of four words from each of three semantic categories (e.g., precious gems, articles of clothing, vegetables, etc.). The participant is asked to listen to the word list read by the examiner and then tries to memorize the words. The word list is read to the participant at the rate of one word every 2 s. Words recalled by the participant are recorded. The same procedure occurs for two more trials. After the third learning trial, the participant is read 24 words. consisting of 12 distractors (items not on the original list) and 12 items that were on the list, and is asked to say “yes” or “no” to identify each word on the original list. The Discrimination Index represents a measure of delayed memory and is calculated by the true positive minus the false positive. The HVLT has many advantages such as the time is takes to administer (10 min), it can be used with moderately and severely cognitively impaired participants and does not appear to have a ceiling effect in normal subjects^25^. Having six versions also allows the HVLT to be used with participants at frequent intervals^26^.

The Trail Making Test (TMT) can identify mild cognitive impairment. The TMT is often used in English-speaking countries because of the use of the English alphabet on the TMT-B. The revised Chinese version was used for measurement in this study^21^. The TMT has two parts (A and B), both of which are timed. The TMTA asks participants to connect the 25 numbers on the paper in order (processing speed). TMTB asks participant to connect the 25 numbers in order with shapes on the page (boxes and circles) to measure executive ability by connecting the circles quickly without lifting the pen from the paper.

### Psychological measures

The Chinese Hospital Anxiety and Depression Scale (HADS) was used to measure depression and anxiety^22^. The HADS consists of 14 self reported items scored from 0 to 3. The anxiety and depression scales are scored on scales of 0–21. Higher total scores indicate higher levels of anxiety and depression. The Chinese version of the HADS has acceptable internal consistency and validity and was evaluated as a reliable tool for the assessment of cancer survivors^22^.

. Psychological assessment was conducted using and Chinese Brief Symptom Inventory (BSI), identifing self-reported psychological symptoms^23^. The Brief Symptom Inventory (BSI) consists of items covering nine symptom areas: somatization, obsession-compulsion, interpersonal sensitivity, depression, anxiety, hostility, phobic anxiety, paranoid ideation and psychoticism; and produces three indicators of distress: global severity index (GSI), Positive symptom distress index (PSDI), and positive symptom total (PST). These indicators measure current or past levels of symptomatology, intensity of symptoms, and the number of reported symptoms. Respondents rank each feeling item (e.g., “your feelings being easily hurt”) on a 5-point scale ranging from 0 (not at all) to 4 (extremely) in terms of any distress over the past 7 days. The GSI is considered the most sensitive indicator of the participant distress. The PST adds the items with non-zero responses and calculates the number of symptoms experienced. The PSDI is the sum of the values of the items receiving non-zero responses divided by the PST. This index provides information about the average level of distress experienced.

### Statistical analysis

Data related to self-reported cognitive and psychological measures were analysed using SPSS for Windows (version 25). Characteristics of the sample were assessed with descriptive statistics including mean (M) and SD or frequency distributions. In comparing subject characteristics, cognitive and psychological measures among three groups used analysis of variance (ANOVA) for continuous variables, and chi-square or Fischer exact tests were used for testing differences in categorical measures. Pearson’s correlation coefficient was used to examine associations between the total score and four subscale scores of the FACT-Cog, with cognitive performance tests and psychological measure scores. To further explore associations between the FACT-Cog subscale scores of PCI and PCA and other measures of cognitive functioning, we obtained partial correlations, controlling for the covariates of age, depressive symptoms.

### Consent to participate

Informed consent was obtained from all individual participants included in the study. No individual data was presented so consent to publish individual data was not required.

## Results

### Sample

A total of 302 women were recruited for the study. There were 106 in Group A (breast cancer survivors with chemotherapy), 100 in Group B (breast cancer survivors with no chemotherapy) and 96 in Group C (non-cancer controls), and these were evenly recruited across the five recruitment centres across China. Table 1 displays demographic and medical information about the sample. See Table 1.

**Table 1:** Study participant characteristics (N = 308).

Characteristic	Total (n = 302)	Group A (n = 106)	Group B (n = 100)	Group C (n = 96)	Chi-square χ^2^	*p*
Age, years						0.107
Mean	49.61	50.25	50.56	47.93		
SD	9.52	9.23	10.05	9.14		
Range	29–75	36–75	29–71	31–73		
BMI						0.026*
Mean	23.23	24	23.22	22.40		
D	3.45	4.05	2.84	3.13		
Range	16.44–38.05	17.22–38.05	17.57–34.45	16.44–35.09		
Living arrangement					5.403	0.067
Living alone	9(3.0%)	1(0.9%)	2(2.0%)	6(6.3%)		
Not living alone	293(97.0%)	105(99.1%)	98(98.0%)	90(93.8%)		
Education					44.048	< 0.01**
No formal education	5(1.7%)	5(4.7%)	0(0.0%)	0(0.0%)		
Low	32(10.6%)	8(7.5%)	13(13.0%)	11(11.5%)		
Middle	142(47.0%)	58(54.7%)	60(60.0%)	24(25.0%)		
High	123(40.7%)	35(33.0%)	27(27.0%)	61(63.5%)		
Work situation					70.583	< 0.01**
No work	93(33.1%)	38(38.8%)	37(40.7%)	18(19.6%)		
Government	11(3.9%)	4(4.1%)	7(7.7.%)	0(0.0%)		
Professionals	70(24.9%)	15(15.3%)	8(8.8%)	47(51.1%)		
Clerks	38(13.5%)	12(12.2%)	11(12.1%)	15(16.3%)		
Farming	28(10.0%)	14(14.3%)	13(14.3%)	1(1.1%)		
Manufacturing	15(5.3%)	4(4.1%)	5(5.5%)	6(6.5%)		
Other	23(8.2%)	9(9.2%)	10(11.0%)	4(4.3%)		
Recruitment Centres						
1	66(21.9%)	26(24,5%)	20(20%)	20(20.8%)		
2	60(19.9%)	20(18.9%)	20(20%)	20(20.8%)		
3	65(21.5%)	20(18.9%)	25(25%)	20(20.8%)		
4	65(21.5%)	20(18.9%)	25(25%)	20(20.8%)		
5	49(16.2%)	20(18.9%)	10(10%)	19(19.8%)		
BSI Score						0.95
Mean	24.82	27.32	27.68	19.09		
SD	26.469	25.795	30.312	21.867		
Range	0–153	0–152	0–153	0–137		
HADS Anxiety						0.499
Mean	3.97	4.42	4.42	3.01		
SD	3.887	4.416	3.856	3.073		
Range	0–20	0–20	0–15	0–12		
HADS Depression						0.120
Mean	3.81	4.31	4.16	2.89		
SD	3.702	4.81	3.72	3.053		
Range	0–18	0–18	0–13	0–13		
Cancer type					12.791	< 0.01**
IDC	171(83.0%)	90(84.9%)	81(81.0%)	N/A		
ILC	6(2.9%)	6(5.7%)	0(0.0%)	N/A		
DCIS	27(13.1%)	8(7.5%)	19(19.0%)	N/A		
Disease stage						
0	27(13.2%)	8(7.5%)	19(19.2%)	N/A	8.134	0.043*
I	7(3.4%)	2(1.9%)	5(5.1%)	N/A		
II	136(66.3%)	76(71.7%)	60(60.6%)	N/A		
III	35(17.1%)	20(18.9%)	15(15.2%)	N/A		
Other complications					36.03	< 0.01**
No	166(80.6%)	69(65.1%)	97(97.0%)	N/A		
AWS, upper arm pain	6(3%)	4(3.7%)	2(2.0%)	N/A		
LYM	34(16.5%)	33(31.1%)	1(1.0%)	N/A		
Radiation therapy					33.6	< 0.01**
No	169(82.0%)	71(67.0%)	98(98.0%)	N/A		
Yes	37(18.0%)	35(33.0%)	2(2.0%)	N/A		
Hormone therapy					24.652	< 0.01**
No	176(85.4%)	78(73.6%)	98(98.0%)	N/A		
Yes	30(14.6%)	28(26.4%)	2(2.0%)	N/A		
Targeted therapy					23.236	< 0.01**
No	184(89.3%)	84(45.7%)	100(100.0%)	N/A		
Yes	22(10.7%)	22(20.8%)	0(0.0%)	N/A		

Data are given as No. (%) unless otherwise noted.

Group A = breast cancer chemotherapy group; Group B = breast cancer group without chemotherapy; Group C = healthy control group.

Low educational level means primary school and junior high school; Middle educational level means high school; High educational level means college and above.

Centres:1, Jing 'an central hospital, Shanghai; 2, Cancer hospital, Yunnan; 3, Central hospital in Zhengzhou; 4, Xiangya hospital in Hunan; 5, Zhuang autonomous region people's hospital in Guangxi Geographical center.

BMI, body mass index; SE, standard error; IDC, invasive ductal carcinoma; ILC, invasive lobular carcinoma; DCIS, ductal carcinoma in situ; AWS, axillary web syndrome; LYM, lymphoedema.

Of the 302 participants included for analysis, the 106 women in Group A were aged 36–75 years (M = 50.25, SE = 9.2), the 100 women in Group B were aged 29–71 years (M = 50.56, SE = 10.1) and the 96 women in Group C were aged 31–73 years (M = 47.93, SE = 9.1). Participants were fairly balanced on age and living arrangements (living alone/not alone), except there were significantly more high school–educated participants in the healthy group compared with the breast cancer groups (*p* < 001). There were more participants with cancer that were not working compared to the healthy controls (*p* < 001). Among the cancer participants, 38.8% of the chemotherapy group (A) and 40.7% of the non-chemotherapy group (B) were not working, compared to 19.6% of the healthy controls. Most breast cancer survivors had invasive ductal carcinoma (IDC), accounting for 83% of participants. Early breast cancer (0–II stage) was most common, accounting for 82.9% of participants. There were significant differences in BMI, living arrangement, education, disease type, symptom stage, other symptoms, radiotherapy, endocrine therapy and radiotherapy among the three groups of participants.

### Comparison of cognitive function (FACT-Cog scores) according to demographic and medical characteristics

The FACT-Cog total scores across groups were compared and results showed that there were statistically significant differences in the FACT-Cog total scores of participants of different ages (*p* = 0.014), living arrangements (*p* = 0.049) and education (*p* = 0.003) (see Table 2). Further examination showed that there were significant differences in the FACT-Cog scores between the middle-aged and older participants (*p* < 0.01). There was also a difference between participants with moderate and low levels of education and those with high levels of education (*p* < 0.01). Participants living alone had higher cognitive impairment.

**Table 2:** Comparison of cognitive function (FACT-Cog scores) according to demographic and medical characteristics.

	FACT-cog total score (M ± SD)	F	*p*
Age		4.41	0.014*
25–35	32.25 ± 24.04		
36–59	25.25 ± 17.09		
60–75	37.57 ± 25.29		
BMI		0.01	0.904
Overweight	28.5 ± 20.49		
Normal	28.2 ± 19.48		
Living arrangements		3.92	0.049*
Lives alone	41.56 ± 18.82		
Not living alone	27.89 ± 20.44		
Education		4.87	0.003*
Uneducated	39.20 ± 35.39		
Low	36.25 ± 24.58		
Middle	30.23 ± 20.76		
High	23.56 ± 17.20		
Cancer type		2.57	0.55
IDC	29.36 ± 21.69		
ILC	17.67 ± 21.94		
DCIS	32.37 ± 22.31		
Disease stage		0.22	0.884
0	32.37 ± 22.30		
I	33.29 ± 12		
II	29.52 ± 23.06		
III	28.66 ± 19.19		
Other complications		1.10	0.359
No	30.32 ± 21.95		
AWS	41.33 ± 22.94		
Left Upper arm pain	45 ± 12.73		
LYM	24.62 ± 22.09		
LYM and AWS	47 ± 0.1		
Radiation therapy		1.09	0.298
No	30.51 ± 22.10		
Yes	26.35 ± 21.34		
Hormone therapy		2.43	0.120
No	30.74 ± 22.77		
Yes	24 ± 15.58		
Targeted therapy		0.10	0.753
No	29.93 ± 22.63		
Yes	28.36 ± 15.67		

Data are given as No. (%) unless otherwise noted.

Group A = breast cancer chemotherapy group; Group B = breast cancer group without chemotherapy; Group C = healthy control group.

Low educational level means primary school and junior high school; Middle educational level means high school; High educational level means college and above.

Centres:1, Jing 'an central hospital, Shanghai; 2, Cancer hospital, Yunnan; 3, Central hospital in Zhengzhou; 4, Xiangya hospital in Hunan; 5, Zhuang autonomous region people's hospital in Guangxi Geographical center.

BMI, Body Mass Index; SE, Standard error; IDC, Invasive ductal carcinoma; ILC, Invasive lobular carcinoma; DCIS, Ductal carcinoma in situ; AWS, Axillary Web Syndrome; LYM, lymphoedema.

### Comparison of cognitive function (FACT-Cog) among cancer survivors and healthy controls

The FACT-Cog results indicated that the total scores and sub-scores of the two cancer groups (chemotherapy and no chemotherapy) were similar and worse than those in the healthy group (see Table 3). There was no significant difference in scores between the two cancer groups. The PCI score of the Group A was significantly worse than the healthy group (*p* = 0.04), and the PCI score of Group B was also significantly worse than the healthy group (*p* = 0.032).

**Table 3:** Comparison of cognitive function outcomes among the three participant groups.

Measures	Mean (SD)/N (%) *			*p*	P (A vs. B)	P (A vs. C)	P (B vs. C)
	Group A (n = 106)	Group B (n = 100)	Group C (n = 96)				
FACT-Cog							
CogPCI	14.48(13.20)	14.69(13.29)	10.95(9.44)	0.055	0.902	0.04*	0.04*
CogOth	1.72(3.28)	1.66(2.69)	1.54(2.43)	0.906	0.886	0.661	0.661
CogPCA	10.80(7.90)	10.13(8.67)	10.20(7.79)	0.807	0.554	0.598	0.598
CogQol	2.83(3.57)	3.21(3.62)	2.48(2.63)	0.306	0.412	0.453	0.453
Total	29.83(22.35)	29.69(21.68)	25.17(16.59)	0.193	0.961	0.107	0.107
HVLT							
Trial 1	4.83(1.93)	4.68(1.72)	5.89(2.04)	< 0.01**	0.571	< 0.01**	< 0.01**
Trial 2	6.84(2.13)	6.50(1.90)	8.03(2.24)	< 0.01**	0.245	< 0.01**	< 0.01**
Trial 3	8.33(2,37)	8.03(2.40)	9.60(2.27)	< 0.01**	0.36	< 0.01**	< 0.01**
Total	20.00(5.74)	19.21(5.24)	23.52(5.75)	< 0.01**	0.311	< 0.01**	< 0.01**
True-positive	10.59(2.14)	10.71(2.09)	10.92(1.58)	0.500	0.681	0.244	0.244
False-positives related	1.52(2.04)	1.27(1.91)	1.28(1.64)	0.565	0.349	0.37	0.37
False-positives unrelated	1.20(2.12)	1.19(2.20)	0.80(1.55)	0.279	0.982	0.158	0.158
Discrimination index	7.88 (4.55)	8.16 (4.86)	8.83(4.11)	0.311	0.654	0.135	0.135
TMT*							
TMTA deficient	17(16.2)	13(13.0)	9(9.6)	0.384	0.519	0.168	0.168
TMTB deficient	5(4.8)	4(4.0)	1(1.1)	0.316	0.802	0.129	0.129

*p < 0.05,**P < 0.01.

A: breast cancer with chemotherapy.

B: breast cancer with no chemotherapy.

C: non-cancer controls.

The HVLT results indicated that rapid memory scores in both groups of breast cancer patients were significantly worse than those in the healthy group (*p* < 0.01). However, there was no significant difference in scores between the two cancer groups (*p* = 0.311) For the delayed memory test component, the accuracy of the two groups of breast cancer patients was lower than that of the healthy group, and the error rate was higher, but the difference was not significant (*p* = 0.311) (see Table 3).

The TMT results showed that the processing speed was worse in the chemotherapy group and the non-chemotherapy group (16.2% and 13%, respectively) compared to the healthy group (9.6%). Executive ability (4.8%) in the chemotherapy group and 4.0% in the non-chemotherapy group was worse than that in the healthy group (1.1%) (see Table 3).

### Correlations between self-reported cognitive function (FACT-Cog) and cognitive performance (HVLT and TMT) for breast cancer participants

The total FACT-COG score, PCI and Oth subscales had a low correlation with performance-based cognition (HVLT and TMT) (r = 0.16–0.20*, p* < 0.01). PCA also had a low correlation with processing speed and executive ability (r = 0.12–0.14, *p* < 0.05)—see Table 4.

**Table 4:** Correlation# between self-rated cognitive function (FACT-Cog) and cognitive performance (HVLT AND TMT) for breast cancer participants.

Measure	Fast memory(HVLT-Total)	Delayed memory (HVLT-discrimination index)	Processing speed(TMT-A)	Executive ability (TMT-B)
FACT-Cog (Total)	− .111	− .154*	.155*	.193**
CogPCI	− .103	− .155*	.146*	.158*
CogOth	− .139*	− .145*	.229**	.296**
CogPCA	− .070	− .055	.081	.127
CogQol	− .026	− .122	.035	.062

#Pearson correlation.

*p < 0.05,**p < 0.01.

### The correlation between cognitive function and psychological status for breast cancer participants

The correlations between FACT-COG overall score and the BSI overall score were strong (r = 0.60 *p* < 0.01), and between the HADS anxiety and depression scales were strong (r = 0.53 and 0.50, *p* < 0.01) indicating that symptom report and anxiety and depression were higher amongst breast cancer survivors reporting cognitive impairment (see Table 5). The correlations between the performance based cognitive tests (HVLT and TMT) and psychological functioning were low, suggesting that processing speed and executive functioning may not be related to anxiety and depression for breast cancer survivors.

**Table 5:** The correlation# between measures of cognitive function and psychological status in breast cancer participants (N = 206).

Measure	BSI Total	GSI	PST	PSDI	1	2	3	4
FACT-Cog Total	.60**	.60**	.60**	.41**	.46**	.69**	.50**	.46**
PCI	.62**	.62**	.60**	.47**	.50**	.72**	.51**	.50**
Oth	.55**	.55**	.50**	.37**	.47**	.63**	.43**	.42**
PCA	.20**	.20**	.30**	.09	.11	.24**	.19**	.17*
Qol	.46**	.46**	.50**	.30**	.34**	.49**	.37**	.40**
HVLT								
Fast memory	− .10**	− .10	− .17	− .19**	− .08*	− .15*	− .07	− .11
Delayed memory	− .11*	− .11	− .14*	− .11	− .50	− .17*	− .08	− .05
TMT								
Processing speed	.20**	.20**	.17*	.17*	.18*	.17*	.19**	.20**
Executive ability	.11	.11	.10	.16*	.05	.15*	.09	.10

Measure	5	6	7	8	9	HADS Anxiety	HADS Depression	
FACT-Cog Total	.50**	.50**	.46**	.48**	.47**	.53**	.50**	
PCI	.50**	.52**	.48**	.49**	.47**	.51**	.46**	
Oth	.46**	.47**	.38**	.50**	.42**	.37**	.37**	
PCA	.14*	.18**	.16*	.17*	.18**	.25**	.27**	
Qol	.48**	.36**	.37**	.32**	.38**	.48**	.42**	
HVLT								
Fast memory	− .10	− .08	− .07	− .02	− .02	− .15*	− .14	
Delayed Memory	− .11	− .18**	− .04	− .09	− .05	− .15*	− .14	
TMT								
Processing speed	.15*	.16*	.12	.14*	.14*	.09	.24**	
Executive ability	.06	.17*	.09	.14*	.08	.00	0.09	

#Pearson correlation

*P < 0.05, **P < 0.01

*GSI* global severity index, *PSDI* positive symptom distress index, *PST* positive symptom total

1, Somatization; 2, Obsession-Compulsion; 3, Interpersonal Sensitivity; 4, Depression; 5, Anxiety; 6, Hostility; 7, Phobic anxiety; 8, Paranoid ideation and 9, Psychoticism

## Discussion

Cognitive changes associated with cancer and cancer treatment are complex. They are also an important issue for cancer survivors^27^. Clinicians have considered cognitive changes to be one of the most persistent late complications of breast cancer treatment^28^ and many clinicians underestimate the impact of cognitive impairments for cancer survivors^29^. Therefore, research on the mechanisms and risk factors of CRCI is important. The main purpose of this study was to explore any links with cognitive dysfunction in breast cancer survivors. This study found that self-reported cognitive function (FACT-Cog) scores of the chemotherapy breast cancer group and the breast cancer group without chemotherapy were higher than those of the healthy group indicating that cognitive function was impacted by breast cancer. However, there was no significant difference in PCI scores between the chemotherapy group and the non-chemotherapy breast cancer group suggesting that chemotherapy may not be a significant factor. The HVLT fast memory and delayed memory scores of participants in both the chemotherapy and non-chemotherapy groups were also significantly worse than that of healthy women which is consistent with previous findings where even long term, cognitive performance can remain significantly worse for breast cancer survivors^30^.

It is important to acknowledge that results indicate that chemotherapy alone may not be a significant factor in the cognitive dysfunction of breast cancer survivors. These results provide some direction for further exploration of the mechanisms of CRCI such as exploring other aspects of breast cancer presentation that could contribute to CRCI. This is consistent with reviews on the role of chemotherapy and cognitive impairment, especially with breast cancer survivors who are also commonly exposed to surgery, aneasthesia, radiotherapy and hormone treatments which can also have cognitive repercussions^31^. This review concluded that it is still uncertain if cognitive impairments are a result of treatment for breast cancer, the cancer itself, or psychological factors^31^.

Breast cancer survivors with lower cognitive performance scores did not necessarily self-report more cognitive problems. This suggests that they were not aware of any changes in cognitive abilities since the diagnosis of cancer, or that they have compensated for their deficits. A lack of a relationship between self-report of cognitive functioning and performance on neuropsychological testing is commonly reported. It may be that breast cancer survivors with CRCI can still perform well on formal performance measures, but it may take a lot more cerebral effort to do so, which they are aware of and report on self-report measures. Breast cancer survivors commonly self-report higher levels of cognitive problems compared to healthy controls that may not be identified on formal testing^32^.

Findings indicated that the FACT-Cog scale and subscale scores including PCI, OTH, PCA, and QOL were associated with fast memory, delayed memory, and executive function. Vardy et al.^33^ investigated the relationship between self-reported cognition and performance-based cognitive assessment. In 29 cases of breast and colorectal cancer, they found no association between them. This study may have been limited by the small sample size. In a recent review article, Hutchison et al.^34^ reviewed 24 studies and found that only eight studies noted a correlation between self-reported cognitive function and performance-based cognitive function, and the results of these studies were inconsistent. The authors speculate that these inconsistencies may be due to the different self-report and performance-based assessment methods used in the studies, and the lack of assessments that use functional activities that are ecologically valid (or based on real-life tasks). Some studies have confirmed the relationship between self-reported cognitive functioning and performance on neuropsychological testing, but few studies in China have jointly assessed both and analysed the correlation between them^34^.

It could be that self-report and performance-based tests of cognitive function assess performance at different periods of time, or even assess different aspects of cognition which may explain inconsistencies^35^. Neuropsychological tests usually assess performance at a single time point, whereas self-report measures assess performance over more extended periods (such as recall over 7 days), and in different settings. Additionally, neuropsychological assessments usually have to be administered in quiet and structured conditions, which is an artificial setting compared to a real-life environment^32^. Therefore, it is recommended that more comprehensive tests be used to detect subtle cognitive impairments in breast cancer survivors.

We conducted a correlation analysis of cognitive function and psychological functioning (as measured by the HADS and BSI) and found that the correlation between self-reported cognitive complaints and psychological functioning for breast cancer survivors was higher than that between performance-based cognitive performance and psychological functioning. This finding is consistent with previous research^36^. There are two possible reasons. One is that the negative emotions caused by breast cancer affect the overall function of the patient, including cognitive function. The other is that the decline of cognitive function is accompanied by the patient's emotional loss. The relationship between the two is unclear. In addition, we used a broader range of emotional problems to analyse the accompanying emotional problems not only anxiety and depression. Obsession-compulsion and depression were highly correlated with self-reported cognitive function.

Finally, we recommend using a multi-factorial model of cognitive decline to ascertain any potential relationships between CRCI and measures of cognitive functioning. We explored the effects of demographics, such as age, and cancer treatment on cognitive function. The study found that age, type of living situation, and level of education were associated with cognitive decline, while emotions as measured by the HADS and BSI also interfered with cognitive functioning.

### Study limitations

Despite being a multi-centre study the participants were mainly from urban areas, so results cannot be generalized to the cognitive function and psychological status of women with different socio-economic backgrounds and those who live in rural areas. As this was a cross-sectional study it was not possible to determine if cognitive impairments detected were persistent or if they improved over time. A future longitudinal study would be able to investigate this further. Breast cancer survivors may be exposed to several drugs with side effects during the 6 months after diagnosis and it is not clear which specific components of chemotherapy amy be related to cognitive changes. Finally, the control group had a higher level of education compared to the treatment groups. This may or may not have had an impact on the cognitive functioning results in this study.

## Conclusion

This study found that cognitive impairments (whether measured by self-report or performance) were associated with Chinese breast cancer survivors compared to healthy controls, however, these cognitive impairments were not always significantly different between breast cancer survivors who had received chemotherapy and those who had not. Cognitive impairments (both self-reported and performance-based) were associated with psychological concerns such as anxiety and depression. It appears that all breast cancer survivors need to be screened for cognitive changes and offered interventions to assist them with everyday functioning. It is unclear if Chinese breast cancer survivors have different experiences of cognitive impairment compared to studies of other populations. Qualitative studies using in-depth interviews would be helpful to understand the experience of participants with a high degree of cognitive impairment and to determine the nature of any psychosocial distress associated with cognitive functioning in this group of breast cancer survivors.

### Supplementary Information

Supplementary Information.

## Data Availability

The datasets generated during and/or analysed during the current study are available from the corresponding author on reasonable request.
