# Supplementary material for: Cognitive impairment experienced by Chinese breast cancer survivors
Source: Sci Rep. 2023 Dec 14;13:22245. doi: 10.1038/s41598-023-49524-0 (PMC10721869; doi:10.1038/s41598-023-49524-0)
Supplement: Supplementary file 1 — Supplementary Information. [file 41598_2023_49524_MOESM1_ESM.pdf]

SURVEY

SOCIODEMOGRAPHIC DETAILS

Age:

Type of cancer diagnosis:

Stage:

Type/s of treatment:

Surgery conducted:

Any side effects of treatment:

Physical changes experienced (TICK):

Hair loss

Changes in body image

Numbness or tingling

Fatigue

Hot flushes

Any other physical changes:

Height:

Weight:

Menopausal status:

How long ago was your last period:

Age at the last period:

Have you been told you are post-menopausal?

Education level (Circle highest qualification level):

No formal schooling

Primary school completion

High school completion

Trade/apprenticeship

Certificate/Diploma

University degree

Higher university degree (Masters, PhD)

Work:

Were you working at diagnosis?

What work did you do at diagnosis?

How many hours per week?

Did you continue to work during treatment?

Did you reduce your working hours?

Did you give up work?

Family support/responsibilities:

What family/other support do you have to help you?:

Are there people that you are responsible for?:

Living arrangements:

Who lives with you (tick all that apply):

No-one – live alone

Partner/spouse

Own children

Someone else's children

Parents

Other adult relatives

Other adults – not family members

Access to healthcare facilities for treatment from home:

What was the travel time for your cancer treatment (chemotherapy, radiation etc)

How did you travel for your cancer treatment? (tick):

Public transport

Own transport

Taxi

Other:

Who have you consulted during your cancer treatment? (tick):

Family doctor

Hospital doctor

Specialist (oncologist)

Allied health professional:

Physiotherapist

Occupational therapist

Dietician

Counsellor

Psychologist

Alternative health practitioner:

Traditional Chinese medicine

Acupuncture

Other:

Financial issues

How do you manage on the income you have available since breast cancer diagnosis and treatment? (Circle one):

It is impossible

It is difficult all the time

It is difficult some of the time

It is not too bad

It is easy

How have you found the cost of breast cancer treatment?:

Brief Symptom Inventory
BSA

"Here I have a list of problems people sometimes have. As I read each one to you, I want you to tell me HOW MUCH THAT PROBLEM HAS DISTRESSED OR BOTHERED YOU DURING THE PAST 7 DAYS INCLUDING TODAY. These are the answers I want you to use. *[Hand card and read answers.]* Do you have any questions?"

0 = Not at all
1 = A little bit
2 = Moderately
3 = Quite a bit
4 = Extremely
R = Refused

DURING THE PAST 7 DAYS, how much were you distressed by:						
1. Nervousness or shakiness inside	0	1	2	3	4	R
2. Faintness or dizziness	0	1	2	3	4	R
3. The idea that someone else can control your thoughts	0	1	2	3	4	R
4. Feeling others are to blame for most of your troubles	0	1	2	3	4	R
5. Trouble remembering things	0	1	2	3	4	R
6. Feeling easily annoyed or irritated	0	1	2	3	4	R
7. Pains in the heart or chest	0	1	2	3	4	R
8. Feeling afraid in open spaces	0	1	2	3	4	R
9. Thoughts of ending your life	0	1	2	3	4	R

DURING THE PAST 7 DAYS, how much were you distressed by:						
10. Feeling that most people cannot be trusted	0	1	2	3	4	R
11. Poor appetite	0	1	2	3	4	R
12. Suddenly scared for no reason	0	1	2	3	4	R
13. Temper outbursts that you could not control	0	1	2	3	4	R
14. Feeling lonely even when you are with people	0	1	2	3	4	R
15. Feeling blocked in getting things done	0	1	2	3	4	R
16. Feeling lonely	0	1	2	3	4	R
17. Feeling blue	0	1	2	3	4	R
18. Feeling no interest in things	0	1	2	3	4	R

0 = Not at all<br>1 = A little bit<br>2 = Moderately<br>3 = Quite a bit<br>4 = Extremely<br>R = Refused

DURING THE PAST 7 DAYS, how much were you distressed by:						
19. Feeling fearful	0	1	2	3	4	R
20. Your feelings being easily hurt	0	1	2	3	4	R
21. Feeling that people are unfriendly or dislike you	0	1	2	3	4	R
22. Feeling inferior to others	0	1	2	3	4	R
23. Nausea or upset stomach	0	1	2	3	4	R
24. Feeling that you are watched or talked about by others	0	1	2	3	4	R
25. Trouble falling asleep	0	1	2	3	4	R
26. Having to check and double check what you do	0	1	2	3	4	R
27. Difficulty making decisions	0	1	2	3	4	R

DURING THE PAST 7 DAYS, how much were you distressed by:						
28. Feeling afraid to travel on buses, subways, or trains	0	1	2	3	4	R
29. Trouble getting your breath	0	1	2	3	4	R
30. Hot or cold spells	0	1	2	3	4	R
31. Having to avoid certain things, places, or activities because they frighten you	0	1	2	3	4	R
32. Your mind going blank	0	1	2	3	4	R
33. Numbness or tingling in parts of your body	0	1	2	3	4	R
34. The idea that you should be punished for your sins	0	1	2	3	4	R
35. Feeling hopeless about the future	0	1	2	3	4	R
36. Trouble concentrating	0	1	2	3	4	R

0 = Not at all
1 = A little bit
2 = Moderately
3 = Quite a bit
4 = Extremely
R = Refused

<u>DURING THE PAST 7 DAYS, how much were you distressed by:</u>

37. Feeling weak in parts of your body	0	1	2	3	4	R
38. Feeling tense or keyed up	0	1	2	3	4	R
39. Thoughts of death or dying	0	1	2	3	4	R
40. Having urges to beat, injure, or harm someone	0	1	2	3	4	R
41. Having urges to break or smash things	0	1	2	3	4	R
42. Feeling very self-conscious with others	0	1	2	3	4	R
43. Feeling uneasy in crowds	0	1	2	3	4	R
44. Never feeling close to another person	0	1	2	3	4	R
45. Spells of terror or panic	0	1	2	3	4	R

<u>DURING THE PAST 7 DAYS, how much were you distressed by:</u>

46. Getting into frequent arguments	0	1	2	3	4	R
47. Feeling nervous when you are left alone	0	1	2	3	4	R
48. Others not giving you proper credit for your achievements	0	1	2	3	4	R
49. Feeling so restless you couldn't sit still	0	1	2	3	4	R
50. Feelings of worthlessness	0	1	2	3	4	R
51. Feeling that people will take advantage of you if you let them	0	1	2	3	4	R
52. Feeling of guilt	0	1	2	3	4	R
53. The idea that something is wrong with your mind	0	1	2	3	4	R

Hospital Anxiety and Depression Scale (HADS)

Tick the box beside the reply that is closest to how you have been feeling in the past week.
Don’t take too long over you replies: your immediate is best.

D	A		D	A	
		**I feel tense or 'wound up':**			**I feel as if I am slowed down:**
	3	Most of the time	3		Nearly all the time
	2	A lot of the time	2		Very often
	1	From time to time, occasionally	1		Sometimes
	0	Not at all	0		Not at all
		**I still enjoy the things I used to enjoy:**			**I get a sort of frightened feeling like 'butterflies' in the stomach:**
0		Definitely as much		0	Not at all
1		Not quite so much		1	Occasionally
2		Only a little		2	Quite Often
3		Hardly at all		3	Very Often
		**I get a sort of frightened feeling as if something awful is about to happen:**			**I have lost interest in my appearance:**
	3	Very definitely and quite badly	3		Definitely
	2	Yes, but not too badly	2		I don't take as much care as I should
	1	A little, but it doesn't worry me	1		I may not take quite as much care
	0	Not at all	0		I take just as much care as ever
		**I can laugh and see the funny side of things:**			**I feel restless as I have to be on the move:**
0		As much as I always could		3	Very much indeed
1		Not quite so much now		2	Quite a lot
2		Definitely not so much now		1	Not very much
3		Not at all		0	Not at all
		**Worrying thoughts go through my mind:**			**I look forward with enjoyment to things:**
	3	A great deal of the time	0		As much as I ever did
	2	A lot of the time	1		Rather less than I used to
	1	From time to time, but not too often	2		Definitely less than I used to
	0	Only occasionally	3		Hardly at all
		**I feel cheerful:**			**I get sudden feelings of panic:**
3		Not at all		3	Very often indeed
2		Not often		2	Quite often
1		Sometimes		1	Not very often
0		Most of the time		0	Not at all
		**I can sit at ease and feel relaxed:**			**I can enjoy a good book or radio or TV program:**
	0	Definitely	0		Often
	1	Usually	1		Sometimes
	2	Not Often	2		Not often
	3	Not at all	3		Very seldom

Please check you have answered all the questions

Scoring:
Total score: Depression (D) ___________ Anxiety (A) ______________
0-7 = Normal
8-10 = Borderline abnormal (borderline case)
11-21 = Abnormal (case)

FACT-Cognitive Function (Version 3)

Below is a list of statements that other people with your condition have said are important. **Please circle or mark one number per line to indicate your response as it applies to the past 7 days.**

	PERCEIVED COGNITIVE IMPAIRMENTS	Never	About once a week	Two to three times a week	Nearly every day	Several times a day
CogA1	I have had trouble forming thoughts	0	1	2	3	4
CogA3	My thinking has been slow..	0	1	2	3	4
CogC7	I have had trouble concentrating	0	1	2	3	4
CogM9	I have had trouble finding my way to a familiar place..	0	1	2	3	4
CogM10	I have had trouble remembering where I put things, like my keys or my wallet	0	1	2	3	4
CogM12	I have had trouble remembering new information, like phone numbers or simple instructions	0	1	2	3	4
CogV13	I have had trouble recalling the name of an object while talking to someone	0	1	2	3	4
CogV15	I have had trouble finding the right word(s) to express myself	0	1	2	3	4
CogV16	I have used the wrong word when I referred to an object	0	1	2	3	4
CogV17b	I have had trouble saying what I mean in conversations with others	0	1	2	3	4
CogF19	I have walked into a room and forgotten what I meant to get or do there..	0	1	2	3	4
CogF23	I have had to work really hard to pay attention or I would make a mistake	0	1	2	3	4
CogF24	I have forgotten names of people soon after being introduced..	0	1	2	3	4

Copyright 2006, 2016

FACT-Cog (Version 3)

Please circle or mark one number per line to indicate your response as it applies to the past 7 days.

		Never	About once a week	Two to three times a week	Nearly every day	Several times a day
CogF25	My reactions in everyday situations have been slow..	0	1	2	3	4
CogC31	I have had to work harder than usual to keep track of what I was doing	0	1	2	3	4
CogC32	My thinking has been slower than usual	0	1	2	3	4
CogC33a	I have had to work harder than usual to express myself clearly	0	1	2	3	4
CogC33c	I have had to use written lists more often than usual so I would not forget things	0	1	2	3	4
CogMT1	I have trouble keeping track of what I am doing if I am interrupted...	0	1	2	3	4
CogMT2	I have trouble shifting back and forth between different activities that require thinking	0	1	2	3	4

Please circle or mark one number per line to indicate your response as it applies to the past 7 days.

	COMMENTS FROM OTHERS	Never	About once a week	Two to three times a week	Nearly every day	Several times a day
CogO1	Other people have told me I seemed to have trouble remembering information	0	1	2	3	4
CogO2	Other people have told me I seemed to have trouble speaking clearly	0	1	2	3	4
CogO3	Other people have told me I seemed to have trouble thinking clearly	0	1	2	3	4
CogO4	Other people have told me I seemed confused	0	1	2	3	4

Copyright 2006, 2016

FACT-Cog (Version 3)

Please circle or mark one number per line to indicate your response as it applies to the past 7 days.

	PERCEIVED COGNITIVE ABILITIES	Not at all	A little bit	Some-what	Quite a bit	Very much
Cog PC1	I have been able to concentrate	0	1	2	3	4
Cog PV1	I have been able to bring to mind words that I wanted to use while talking to someone	0	1	2	3	4
Cog PM1	I have been able to remember things, like where I left my keys or wallet	0	1	2	3	4
Cog PM2	I have been able to remember to do things, like take medicine or buy something I needed	0	1	2	3	4
Cog PF1	I am able to pay attention and keep track of what I am doing without extra effort	0	1	2	3	4
Cog PCH 1	My mind is as sharp as it has always been	0	1	2	3	4
Cog PCH 2	My memory is as good as it has always been	0	1	2	3	4
Cog PMT 1	I am able to shift back and forth between two activities that require thinking	0	1	2	3	4
Cog PMT 2	I am able to keep track of what I am doing, even if I am interrupted	0	1	2	3	4

Please circle or mark one number per line to indicate your response as it applies to the past 7 days.

	IMPACT ON QUALITY OF LIFE	Not at all	A little bit	Some-what	Quite a bit	Very much
CogQ35	I have been upset about these problems	0	1	2	3	4
CogQ37	These problems have interfered with my ability to work	0	1	2	3	4
CogQ38	These problems have interfered with my ability to do things I enjoy	0	1	2	3	4
CogQ41	These problems have interfered with the quality of my life	0	1	2	3	4

Copyright 2006, 2016

HOPKINS VERBAL LEARNING TEST

Form 1: four-legged animals, precious stones, human dwellings

Part A: Free Recall

	Trial 1	Trial 2	Trial 3
EMERALD	________	________	________
HORSE	________	________	________
TENT	________	________	________
SAPPHIRE	________	________	________
HOTEL	________	________	________
CAVE	________	________	________
OPAL	________	________	________
TIGER	________	________	________
PEARL	________	________	________
COW	________	________	________
HUT	________	________	________
# CORRECT	________	________	________

HORSE	ruby*	CAVE	balloon	coffee	LION
house*	OPAL	TIGER	boat	scarf	PEARL
HUT	EMERALD	SAPPHIRE	dog*	apartment*	penny
TENT	mountain	cat*	HOTEL	COW	diamond*

Part B: Recognition:

# True-Positives:______________/12

# False-Positive Errors: Related:____________/6 Unrelated:__________/6

Discrimination Index: (# True-Positives) – (# False-Positives) = ________________

HOPKINS VERBAL LEARNING TEST
Form 2: kitchen utensils, alcoholic beverages, weapons

Part A: Free Recall

	Trial 1	Trial 2	Trial 3
FORK	________	________	________
RUM	________	________	________
PAN	________	________	________
PISTOL	________	________	________
SWORD	________	________	________
SPATULA	________	________	________
BOURBON	________	________	________
VODKA	________	________	________
POT	________	________	________
COW	________	________	________
HUT	________	________	________
WINE	________	________	________
# CORRECT	________	________	________

Part B: Recognition:

spoon*	PISTOL	doll	whiskey*	FORK	POT
harmonica	can opener*	SWORD	pencil	gun*	VODKA
knife*	RUM	trout	BOMB	PAN	gold
WINE	lemon	SPATULA	BOURBON	beer*	RIFLE

True-Positives:_____________/12

False-Positive Errors: Related:___________/6 Unrelated:__________/6

Discrimination Index: (# True-Positives) – (# False-Positives) = _______________

HOPKINS VERBAL LEARNING TEST
Form 3: musical instruments, fuels, food flavorings

Part A: Free Recall

	Trial 1	Trial 2	Trial 3
SUGAR	________	________	________
TRUMPET	________	________	________
VIOLIN	________	________	________
COAL	________	________	________
GARLIC	________	________	________
KEROSINE	________	________	________
VANILLA	________	________	________
WOOD	________	________	________
CLARINET	________	________	________
FLUTE	________	________	________
CINNAMON	________	________	________
GASOLINE	________	________	________
# CORRECT	________	________	________

Part B: Recognition:

pepper*	GARLIC	WOOD	drum*	oil*	SUGAR
Harmonica	salt*	priest	chair	COAL	CLARINET
TRUMPET	basement	CINNAMON	FLUTE	electricity*	Moon
KEROSINE	VANILLA	GASOLINE	sand	piano*	VIOLIN

# True-Positives:______________/12

# False-Positive Errors: Related:____________/6 Unrelated:__________/6

Discrimination Index: (# True-Positives) – (# False-Positives) = ________________

HOPKINS VERBAL LEARNING TEST
Form 4: birds, articles of clothing, carpenter's tools

Part A: Free Recall

	Trial 1	Trial 2	Trial 3
CANARY	______	______	______
SHOES	______	______	______
EAGLE	______	______	______
BLOUSE	______	______	______
NAILS	______	______	______
CROW	______	______	______
BLUEBIRD	______	______	______
SCREWDRIVER	______	______	______
PANTS	______	______	______
CHISEL	______	______	______
SKIRT	______	______	______
WRENCH	______	______	______
# CORRECT	______	______	______

Part B: Recognition:

BLUEBIRD	shirt*	CHISEL	EAGLE	chocolate	robin*
chapel	SCREWDRIVER	CROW	sparrow*	WRENCH	PANTS
NAILS	socks*	child	SHOES	hair	hammer*
CANARY	apple	SKIRT	saw*	silver	BLOUSE

True-Positives:______________/12

False-Positive Errors: Related:____________/6 Unrelated:___________/6

Discrimination Index: (# True-Positives) – (# False-Positives) = ________________

HOPKINS VERBAL LEARNING TEST

Form 5: occupations/professions, sports, vegetables

Part A: Free Recall

	Trial 1	Trial 2	Trial 3
TEACHER	______	______	______
BASKETBALL	______	______	______
LETTUCE	______	______	______
DENTIST	______	______	______
TENNIS	______	______	______
BEAN	______	______	______
ENGINEER	______	______	______
POTATO	______	______	______
PROFESSOR	______	______	______
GOLF	______	______	______
CORN	______	______	______
SOCCER	______	______	______
# CORRECT	______	______	______

Part B: Recognition:

True-Positives:_____________/12

TENNIS	football*	PROFESSOR	spinach*	lawyer*	submarine
GOLF	DENTIST	LETTUCE	spider	water	BEAN
BASKETBALL	doctor*	CORN	baseball*	TEACHER	snake
carrot*	ENGINEER	glove	SOCCER	POTATO	tulip

False-Positive Errors: Related:___________/6 Unrelated:__________/6

Discrimination Index: (# True-Positives) – (# False-Positives) = _______________

HOPKINS VERBAL LEARNING TEST
Form 6: fish, parts of a building, phenomens

Part A: Free Recall

	Trial 1	Trial 2	Trial 3
SHARK	________	________	________
WALL	________	________	________
HERRING	________	________	________
RAIN	________	________	________
FLOOR	________	________	________
HAIL	________	________	________
CATFISH	________	________	________
ROOF	________	________	________
SALMON	________	________	________
STORM	________	________	________
CEILING	________	________	________
SNOW	________	________	________
# CORRECT	________	________	________

Part B: Recognition:

True-Positives:______________/12

HAIL	bass*	SNOW	bank	FLOOR	mustard
window*	CEILING	canyon	RAIN	ladder	STORM
HERRING	SALMON	tornado*	trout*	melon	ROOF
SHARK	hurricane*	elbow	CATFISH	WALL	door*

False-Positive Errors: Related:___________/6 Unrelated:__________/6

Discrimination Index: (# True-Positives) – (# False-Positives) = _______________

Trail Making Test (TMT) Parts A & B

Instructions:

Both parts of the Trail Making Test consist of 25 circles distributed over a sheet of paper. In Part A, the circles are numbered 1 – 25, and the patient should draw lines to connect the numbers in ascending order. In Part B, the circles include both numbers (1 – 13) and letters (A – L); as in Part A, the patient draws lines to connect the circles in an ascending pattern, but with the added task of alternating between the numbers and letters (i.e., 1-A-2-B-3-C, etc.). The patient should be instructed to connect the circles as quickly as possible, without lifting the pen or pencil from the paper. Time the patient as he or she connects the "trail." If the patient makes an error, point it out immediately and allow the patient to correct it. Errors affect the patient's score only in that the correction of errors is included in the completion time for the task. It is unnecessary to continue the test if the patient has not completed both parts after five minutes have elapsed.

Step 1: Give the patient a copy of the Trail Making Test Part A worksheet and a pen or pencil.

Step 2: Demonstrate the test to the patient using the sample sheet (Trail Making Part A – *SAMPLE*).

Step 3: Time the patient as he or she follows the "trail" made by the numbers on the test.

Step 4: Record the time.

Step 5: Repeat the procedure for Trail Making Test Part B.

Scoring:

Results for both TMT A and B are reported as the number of seconds required to complete the task; therefore, higher scores reveal greater impairment.

	**Average**	**Deficient**	**Rule of Thumb**
**Trail A**	29 seconds	> 78 seconds	Most in 90 seconds
**Trail B**	75 seconds	> 273 seconds	Most in 3 minutes

Sources:

- Corrigan JD, Hinkeldey MS. Relationships between parts A and B of the Trail Making Test. *J Clin Psychol.* 1987;43(4):402–409.
- Gaudino EA, Geisler MW, Squires NK. Construct validity in the Trail Making Test: what makes Part B harder? *J Clin Exp Neuropsychol.* 1995;17(4):529-535.
- Lezak MD, Howieson DB, Loring DW. *Neuropsychological Assessment.* 4th ed. New York: Oxford University Press; 2004.
- Reitan RM. Validity of the Trail Making test as an indicator of organic brain damage. *Percept Mot Skills.* 1958;8:271-276.

TRAIL MAKING

Part A

SAMPLE

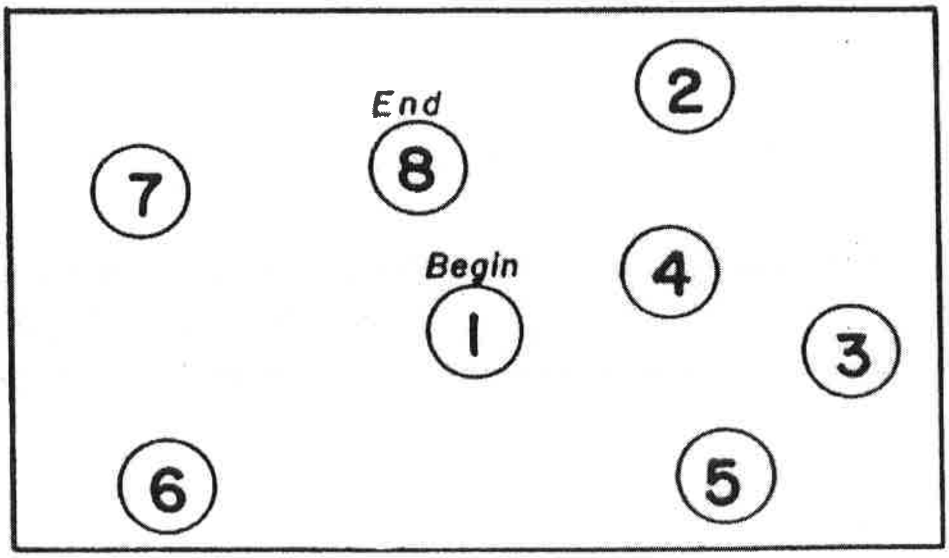

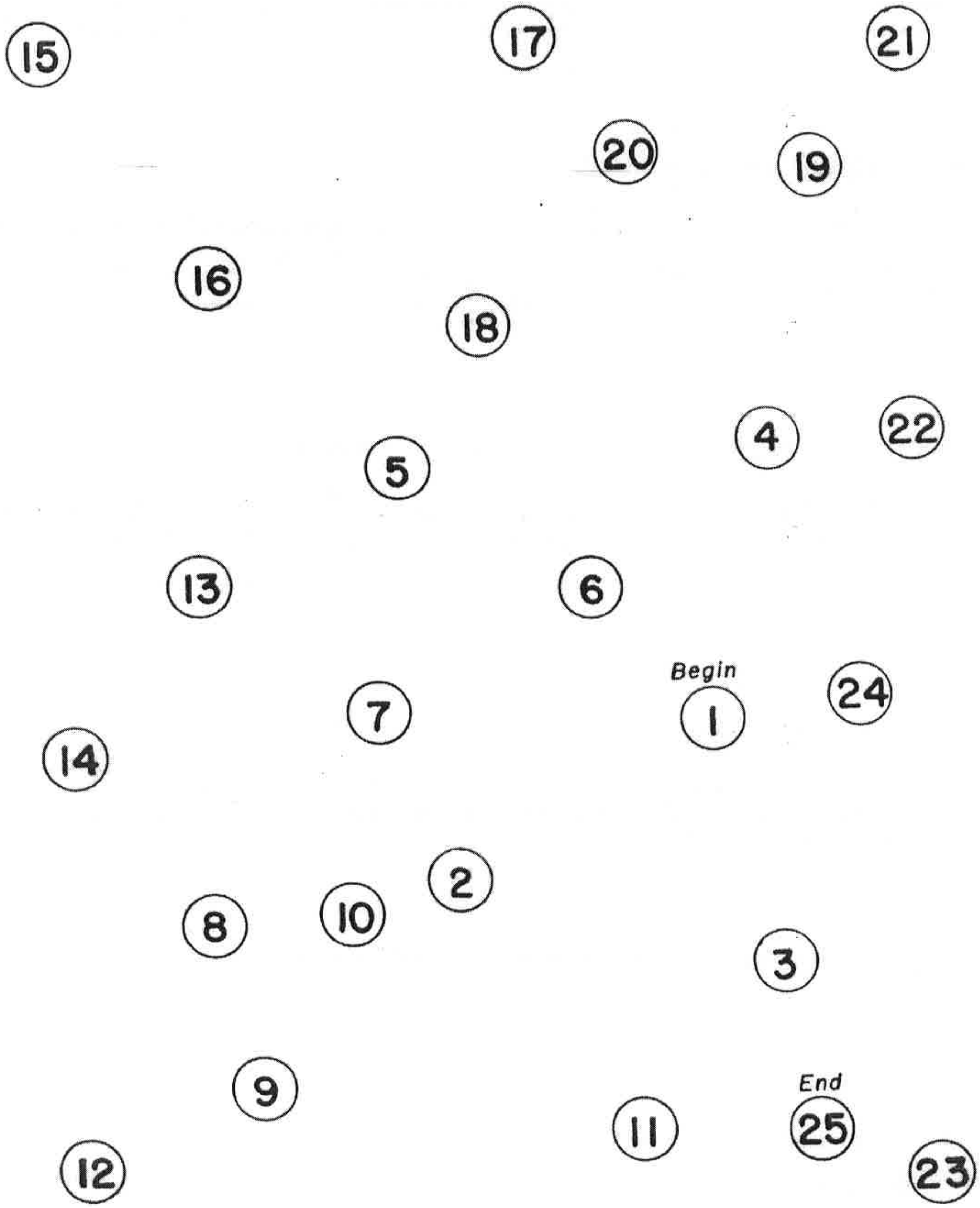
15
17
21
20
19
16
18
4
22
5
13
6
Begin
1
24
7
14
2
10
8
3
9
End
25
11
23
12

TRAIL MAKING

Part B

SAMPLE

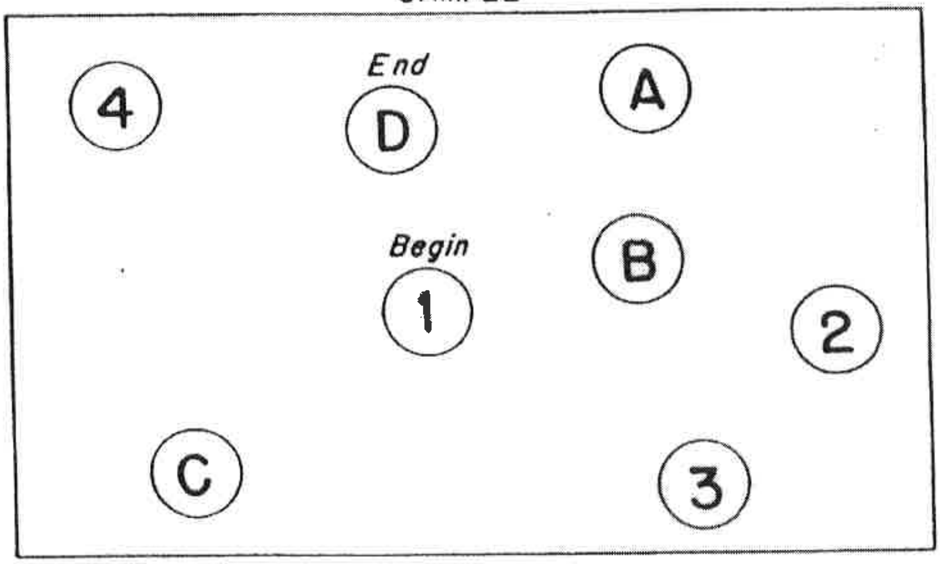

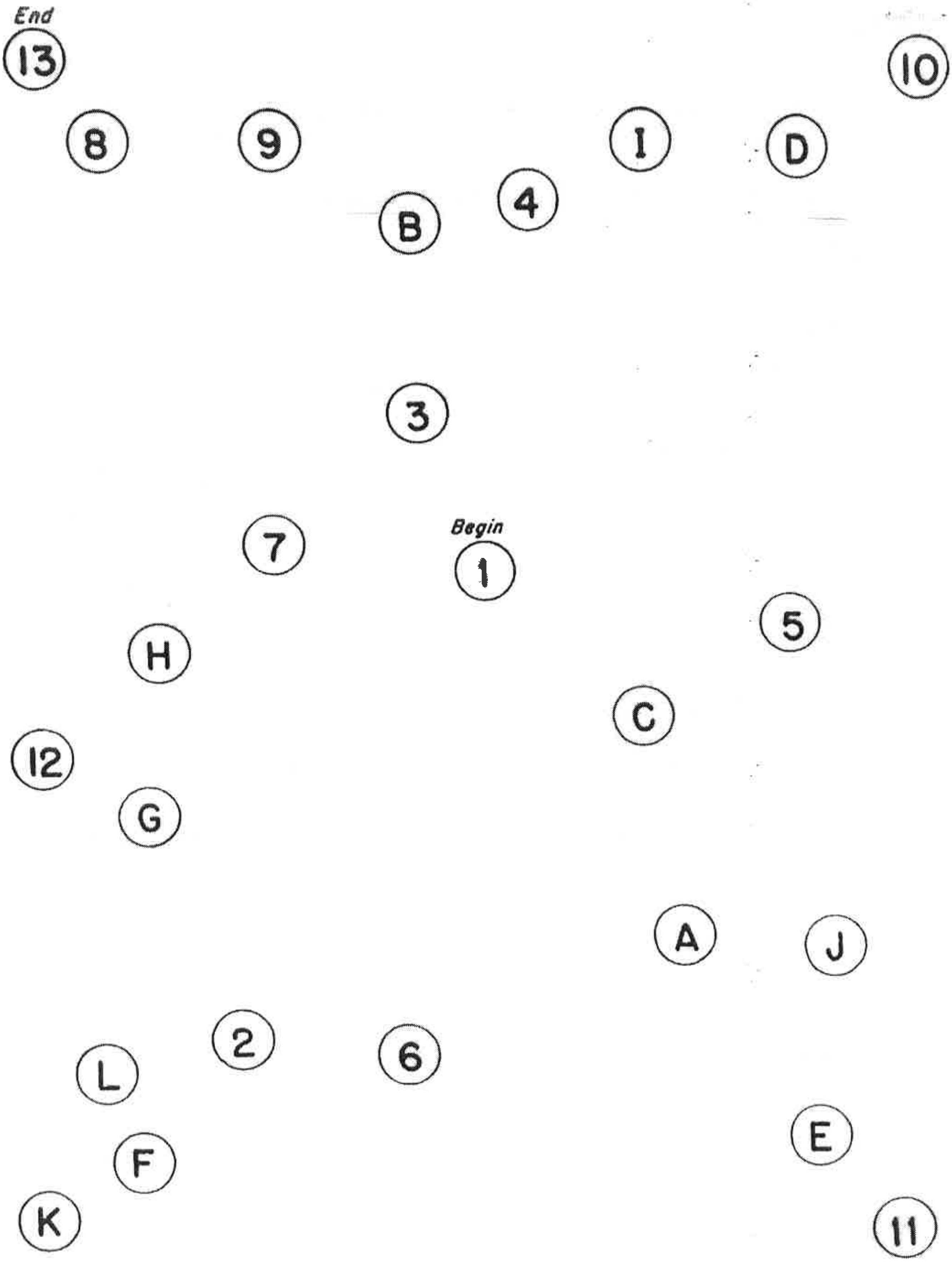
End
13
10
8
9
I
D
4
B
3
Begin
7
1
5
H
C
12
G
A
J
2
6
L
E
F
K
11
